# Current insights into fungal species diversity and perspective on naming the environmental DNA sequences of fungi

**DOI:** 10.1080/21501203.2019.1614106

**Published:** 2019-05-07

**Authors:** Bing Wu, Muzammil Hussain, Weiwei Zhang, Marc Stadler, Xingzhong Liu, Meichun Xiang

**Affiliations:** a State Key Laboratory of Mycology, Institute of Microbiology, Chinese Academy of Sciences, Beijing, China; b Department Microbial Drugs, Helmholtz Centre for Infection Research, Braunschweig, Germany; c University of Chinese Academy of Sciences, Beijing, China

**Keywords:** Fungi, biodiversity, molecular methods, numbers of fungi, fungal phylogeny

## Abstract

The global bio-diversity of fungi has been extensively investigated and their species number has been estimated. Notably, the development of molecular phylogeny has revealed an unexpected fungal diversity and utilisation of culture-independent approaches including high-throughput amplicon sequencing has dramatically increased number of fungal operational taxonomic units. A number of novel taxa including new divisions, classes, orders and new families have been established in last decade. Many cryptic species were identified by molecular phylogeny. Based on recently generated data from culture-dependent and -independent survey on same samples, the fungal species on the earth were estimated to be 12 (11.7–13.2) million compared to 2.2–3.8 million species recently estimated by a variety of the estimation techniques. Moreover, it has been speculated that the current use of high-throughput sequencing techniques would reveal an even higher diversity than our current estimation. Recently, the formal classification of environmental sequences and permission of DNA sequence data as fungal names’ type were proposed but strongly objected by the mycologist community. Surveys on fungi in unusual niches have indicated that many previously regarded “unculturable fungi” could be cultured on certain substrates under specific conditions. Moreover, the high-throughput amplicon sequencing, shotgun metagenomics and a single-cell genomics could be a powerful means to detect novel taxa. Here, we propose to separate the fungal types into physical type based on specimen, genome DNA (gDNA) type based on complete genome sequence of culturable and uncluturable fungal specimen and digital type based on environmental DNA sequence data. The physical and gDNA type should have priority, while the digital type can be temporal supplementary before the physical type and gDNA type being available. The fungal name based on the “digital type” could be assigned as the “clade” name + species name. The “clade” name could be the name of genus, family or order, etc. which the sequence of digital type affiliates to. Facilitating future cultivation efforts should be encouraged. Also, with the advancement in knowledge of fungi inhabiting various environments mostly because of rapid development of new detection technologies, more information should be expected for fungal diversity on our planet.

## Why should we estimate the fungal diversity?

Fungi are the second most species-rich organism group after the insects (Purvis and Hector 2000); hence, it is more challenging to complete the global fungal inventory, as compared to other organisms such as plants. Fungi play key roles in ecosystems as decomposers, mutualists and pathogens, while in most cases, role of individual fungus in nature is still unknown (Schmit and Mueller 2007). The increasing number of virulent infectious diseases caused by fungi is regarded as a worldwide threat to food security (Hyde et al. 2018a). An unprecedented number of diseases caused by fungi and fungal-like organisms (e.g. oomycetes) have recently resulted in some of the most severe die-offs and extinctions ever witnessed in wild species (Fisher et al. 2012). Among these incidences, most of the pathogenic fungi were previously undescribed or very little information was available on them before the disasters occurred (Blehert et al. 2009; Farrer et al. 2011). Considering this, the description of all fungal species can help humankind to identify, guard and prevent disasters incurred by fungal pathogens.

**Figure 1.**
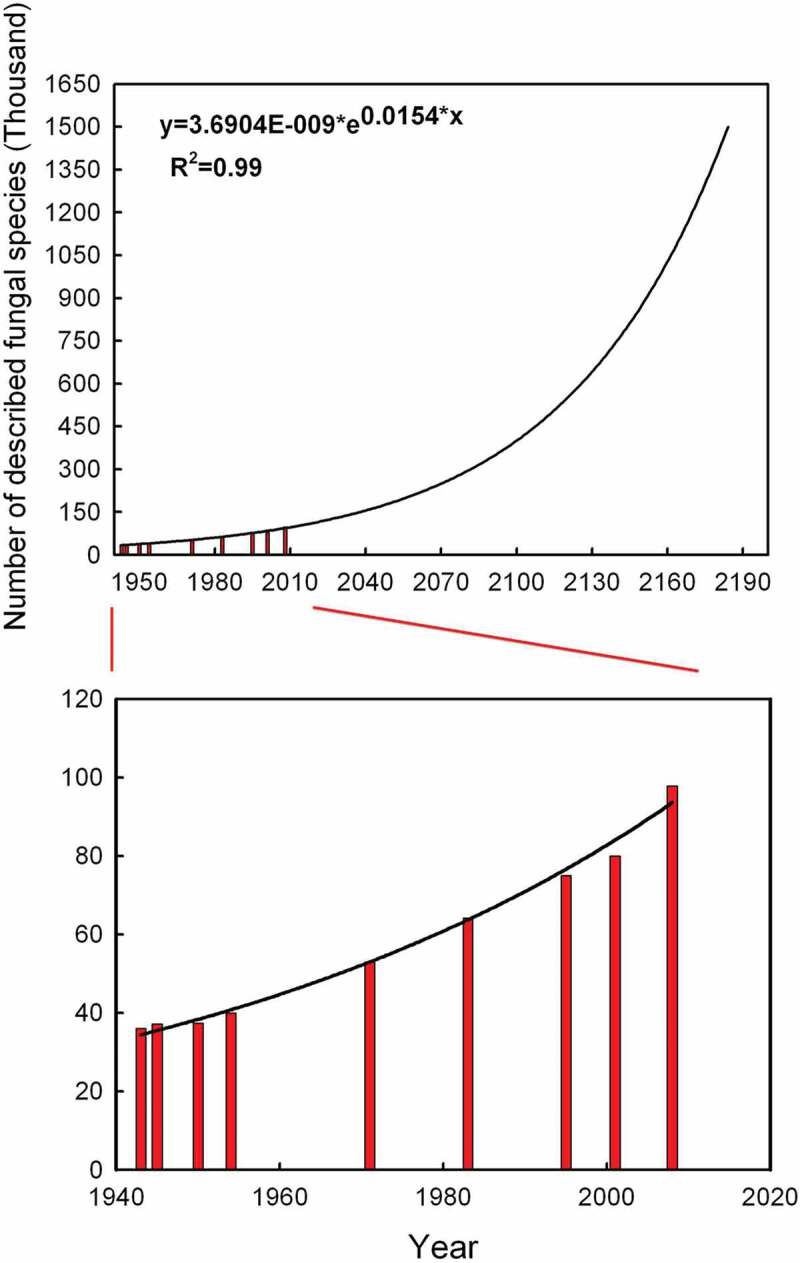
The regression relationship between time and described fungal species using Sigma State software.

It has been estimated that all the plant, animal or microbial species would be described in about 30–90 years before they become extinct, considering that there are probably 1.5–3 million undescribed species on the earth with an extinction rates of 0.01–1% (at most 5%) per decade (Costello et al. 2013). To date, completely described fungi accounted for only 7% of the 1.5 million species hypothesis, i.e. a relatively conserved estimate (Hawksworth 2004). Average numbers of species newly described per year based on every decade evaluation were 1229 from 1980 to 1989, 1097 from 1990 to 1999 (Hawksworth 2001) and 1196 from 1999 to 2009 (Hibbett et al. 2011). A calculation indicated that the average numbers of new species increased to 1430 each year from 2008 to 2012 (Dai et al. 2015). However, it was assumed that the number of fungal species ranged between 3.5 and 5.1 million based on next-generation sequencing (Blackwell 2011). In contrast, an updated estimate of fungal diversity showed that the fungal species ranged from 2.2 to 3.8 million worldwide (Hawksworth and Luecking 2017). Here, a model was constructed to indicate the description rate of fungi through the Sigma State software (Sigma State 3.5. SPSS, USA). Numbers of known fungi from the series editions of “Dictionary of the Fungi” were taken into consideration; there was an exponential regression relationship between described fungal numbers and years (*R*^2^ = 0.99, *p* < 0.0001) (Figure 1). Based on this regression analysis, 1.5 million fungal species estimated by Hawksworth (1991) could be described only by the year 2184. Similarly, the estimates of 2.2 and 3.8 million fungal species could be described by the years 2210 and 2245, respectively. Besides, it is important that data on biogeographic distributions, levels of endemism and host specificity must be taken into account when estimating the global fungal diversity (Mueller and Schmit 2007). The above methods were hampered by the fact that all the data and estimates are based on ITS nrDNA sequence data and it is now well known that this DNA locus is not well suited to reflect the true species diversity within a given genus or family (Hongsanan et al. 2018). On the other hand, recent, intensive studies based on comprehensive inventories of certain fungal genera and families have demonstrated that in countries and areas that were hitherto neglected by mycological taxonomists, up to over 90% of the collected specimens may constitute undescribed species (Hyde et al. 2018b).

## Previous estimates of fungal diversity

Estimations of the total number of fungi have major implications for systematics, resources and classification (Hawksworth 1991). The earliest estimates were primarily based on the numbers of fungi recorded on particular plants. The number of fungi was estimated to be about 100,000 by Bisby and Ainsworth (1943), 250,000 by Martin (1951) and 1.5 million by Hawksworth (1991). The latter has been most widely accepted for two decades (Table 1). Recently, it was hypothesised that the current estimated fungal species range from 3.5 to 5.1 million, worldwide (Blackwell 2011). However, there are many fungi that do not have an apparent host-specificity and are rather ubiquitous. For instance, *Daldinia eschscholtzii* is one of the most frequently encountered endophytes in subtropical and tropical areas and its stromata have been found in numerous countries around the world (Stadler et al. 2014; Helaly et al. 2018). This fungus can also occur as endophyte of marine algae (Tarman et al. 2012), as endosymbiont of mantis gut (Zhang et al. 2011) and as contaminant of human blood culture (Chan et al. 2015). Another fungus *Exophiala alcalophila* was first derived from soil, and later was found in soap container, bath water and even mildly symptomatic human skin (de Hoog et al. 2011). These and many other examples depict that concise estimates on the actual number of fungal species can only be made, once we have achieved thorough understanding on their ecology and life strategies.

**Table 1.** Estimations on the global number of fungal species.

Estimated species (million)	Tips	Literatures
0.1		Bisby and Ainsworth 1943
0.25		Martin 1951
2.7		Pascoe 1990
1.5		Hawksworth 1991
1		Hammond 1992
1	On tropical plants	Smith and Waller 1992
1.5	Insect fungi	Hywel-Jones 1993
1		Rossman 1994
1.3	Endophytes	Dreyfuss and Chapela 1994
1.5		Hammond 1995
0.27	Plant pathogens	Shivas and Hyde 1997
0.04–0.07	World ascomycetes	Aptroot 2001
9.9		Cannon 1997
0.2	Mexico	Guzman 1998
More than 1.5	Very conservative	Fröhlich and Hyde 1999
0.5		May 2000
More than 1.5		Arnold et al. 2000
2.3		Hawksworth 2001
0.06	Ascomycota	de Meeûs and Renaud 2002
0.025	Basidiomycota	de Meeûs and Renaud 2002
3.5–5.1		O’Brien et al. 2005
0.17	South Africa	Crous et al. 2006
0.72		Schmit and Mueller 2007
0.18	China	Dai and Zhuang 2010
5.1		Blackwell 2011
0.61	Land	Mora et al. 2011
0.005	Ocean	Mora et al. 2011
1.5–3		Hawksworth 2012
2.2–3.8		Hawksworth and Luecking 2017

Of course, divergent opinions to this are also available. Based on the information in the US National Fungus Collection database, Rossman (1994) estimated the number of fungi to be just over 1 million by estimating the number of fungi by taxonomic group. Based on the personal experience and other published studies, Dreyfuss and Chapela (1994) estimated that 1.3 million endophytic fungi alone still await discovery.

The major limitation of these estimates is that they only target the fungi that either produce fruiting bodies (can be identified upon microscopic examination) or can be easily cultured on artificial media (Duong et al. 2006). Many endophytes do not sporulate in culture (White and Cole 1986), making visual identification of some endophytic cultures challenging. Direct morphological examination of fruiting structures on substrates or media may introduce biasness in estimating fungal diversity (Guo et al. 2001; Promputtha et al. 2004). Among fungal species in body and gut samples, only 10 operational taxonomic units (OTUs) were found to be shared between them and 58.7% of them were singletons, i.e. found only once (Anslan et al. 2016). When considering the fungal diversity in 20 wetlands in China, of the 177 species, 65 were isolated only once; 40 were found in only two or three locations; and 89 were endemic (Wu et al. 2013). Although “everything is everywhere”, the distribution of fungal species is distinct. It is also probable that fungal species number could be much higher than the current revised estimates of 2.2–3.8 million. Only the insect fungi were estimated to be 1.5 million by Hywel-Jones (1993).

The emergence of more and more uncultured fungi indicated that the diversity of fungal species was generally underestimated. The development of molecular techniques, such as high-throughput sequencing, has contributed tremendously in identification of previously unknown diversity. For example, the site-dependent detections based on the ratio of the fungal numbers revealed by high-throughput sequencing to the plant species number indicated a high rate of new species accumulation and an estimate of 3.5–5.1 million species of fungi (O’Brien et al. 2005).

## Revised estimate of fungal diversity

The number of fungal species on the planet was estimated from the data of published literatures to compare fungal species numbers by culture-dependent methods and culture-independent approaches from same samples. The OTUs were detected by culture-independent approaches including TGGE (Thermal Gradient Gel Electrophoresis), DGGE (Denaturing Gradient Gel Electrophoresis), SSCP (Single-Strand Conformation Polymorphism), RFLP (Restriction Fragment Length Polymorphism), TRFLP (Terminal Restriction Fragment Length Polymorphism), ARDRA (Amplified Ribosomal DNA Restriction Analysis), 454 Pyrosequencing and Illumina MiSeq sequencing. Although these methods can hardly provide species-specific information, the high numbers of detected OTUs revealed an enormous, unprecedented magnitude of fungal diversity. The literature survey revealed a ratio of cultured fungal numbers to OTUs as 1:0.6–1:107.3 according to different culture-independent methods, with an average ratio as 1:8.8. Considering their overlaps (Table 2), the total fungal estimation should be 7.8–8.8 times that of culture-dependent methods. Based on the widely accepted estimate of 1.5 million culturable fungal species (Hawksworth 1991) and then 2.2–3.8 million (Hawksworth and Luecking 2017), our estimation range of total fungal diversity is about 12 million (11.7–13.2) species.

**Table 2.** Comparison of fungal species numbers resulted by culture-dependent and -independent methods.

Culture-independent methods	Substrates	Species by culture	OTU numbers	Ratio	References
TGGE	Wheat rhizosphere	30	41	1.4	Smit et al. 1999
TGGE	Air	24	20	0.8	Nieguitsila et al. 2007
DGGE	Plant hair roots	38	32	0.8	Bougoure and Cairney 2005
DGGE	Plant hair roots	22	24	1.1	Bougoure and Cairney 2005
DGGE	Plant hair roots	25	30	1.2	Bougoure and Cairney 2005
DGGE	Soil	71	100	1.4	Arenz et al. 2006
DGGE	Soil	37	43	1.2	Malosso et al. 2006
DGGE	Sponge	14	23	1.6	Gao et al. 2008
DGGE	Sponge	20	21	1.1	Gao et al. 2008
DGGE	Acidic soil	5	35–40	8	Cosgrove et al. 2010
DGGE	Neutral soil	4	35–40	10	Cosgrove et al. 2010
DGGE	Deep sea sediment	19	46	2.4	Singh et al. 2012
DGGE	Periglacial soil	37	75	2.0	Rodolfi et al. 2016
DGGE	Dough fermentation starter	4	16	4.0	Li et al. 2016
DGGE	Traditionally prepared dried starters	19	46	2.4	Sha et al. 2018
DGGE	Book	7	24	3.4	Okpalanozie et al. 2018
SSCP	Soil	21	42	2.0	Zachow et al. 2009
SSCP	*Ophiocordyceps sinensis*	92	118	1.3	Zhang et al. 2010
RFLP	Soil	29	30	1.0	Viaud et al. 2000
RFLP	Mycorrhizal roots	39	156	4.0	Allen et al. 2003
RFLP	Adult date palm	5	13	2.6	Chobba et al. 2013
RFLP	Needle litter	71	122	1.7	Haňáčková et al. 2015
T-RFLP	Soil	12	85	7.1	Lord et al. 2002
T-RFLP	Soil	12	23 (18S)	1.9	Lord et al. 2002
18S ARDRA	Grassland soils	6	18	3.0	Hunt et al. 2004
18S ARDRA	Grassland soils	7	22	3.1	Hunt et al. 2004
18S ARDRA	Grassland soils	8	29	3.6	Hunt et al. 2004
ARDRA	Soil	36	67	1.9	Malosso et al. 2006
Sequencing	Soil (Orbiliaceae)	8	18	2.3	Smith and Jaffee 2009
PCR-sequencing	Human gut	5	18	3.6	Gouba et al. 2013
Quantitative PCR	Dust	35	450	12.9	Nonnenmann et al. 2012
RISA	Rice wine wheat Qu	8	5	0.6	Xie et al. 2007
Clone libraries	Human distal gut	3	13	4.3	Scanlan and Marchesi 2008
Clone libraries	Dust	35	394	11.3	Pitkäranta et al. 2008
Clone libraries	Moisture buildings	33	305	9.2	Pitkäranta et al. 2011
Clone libraries	Deep sea sediment	20	39	2.0	Singh et al. 2011
Clone libraries	Root	153	304	2.0	Walker et al. 2011
Clone libraries	Human faecal	5	16	3.2	Hamad et al. 2016
Clone libraries	Epoxy resin	16	25	1.6	Pangallo et al. 2015
Clone libraries	Cheese	8	17	2.1	Šuranská et al. 2016
Pyrosequencing	Grassland	7	74	10.6	Lumini et al. 2010
454 pyrosequencing	Root	39	312	8.0	Tedersoo et al. 2010
454 pyrosequencing	Air	24	986	41.1	Adams et al. 2013
454 pyrosequencing	Soil	29	54	1.9	Hirsch et al. 2013
454 pyrosequencing	Hydrocarbon-contaminated soils	49	360	7.3	Stefani et al. 2015
454 pyrosequencing	Plant roots	41	592	14.4	Bourdel et al. 2016
454 pyrosequencing	Grape must	5	15	3.0	Wang et al. 2015a
454 pyrosequencing	Zea mays	9	60	6.7	Bokati et al. 2016
454 pyrosequencing	*Triticum aestivum*	18	248	13.8	Bokati et al. 2016
454 pyrosequencing	Beer	18	1931	107.3	Takahashi et al. 2015
454 pyrosequencing	Chicha	16	81	5.1	Mendoza et al. 2017
454 pyrosequencing	Must	10	387	38.7	David et al. 2014
Illumina MiSeq	Root	43	1168	27.2	Parmar et al. 2018
Illumina MiSeq	Book	13	179	13.8	Kraková et al. 2018
Illumina MiSeq	Leaf	70	597	8.5	Siddique et al. 2017
Illumina MiSeq	Cheese	9	14	1.6	Santos et al. 2017
Illumina MiSeq	Wine	28	254	9.1	Li et al. 2018
Illumina MiSeq	Collembola body	31	896	28.9	Anslan et al. 2016
Illumina MiSeq	Collembola gut	25	667	26.7	Anslan et al. 2016
Illumina MiSeq	Rhizospheric	43	860	20.0	Miao et al. 2016
Illumina MiSeq	Chronic-wound	17	482	28.4	Kalan et al. 2016
Illumina HiSeq2500	Stems of grapevine	28	59	2.1	Dissanayake et al. 2018
Illumina HiSeq2500	Museum	9	85	9.4	Liu et al. 2018
Illumina HiSeq2501	Lake	398	479	1.2	Wahl et al. 2018
	**Average ratio**			**8.8**	

Although more fungal species were detected by culture-independent approaches than that of culture-dependent methods, the fungal species detected by both approaches are not actually overlapping, even for the dominant fungal species. A case study on the mycobiota of naturally occurring *Ophiocordyceps sinensis* specimens (including stromata, sclerotia and the complex of mycelial cortices and attached soil particles outside the sclerotia) revealed 118 unique OTUs identified by SSCP from three samples vs. 98 species from diverse samples by culture-dependent methods. However, out of 92 cultured fungal taxa and 118 OTUs detected by the SSCP method, only 13 OTUs were detected by both methods (Zhang et al. 2010). Less symmetry between data obtained from different methods was also reported in many other cases (Zhang et al. 2009; Avis et al. 2010). There might be two reasons: on one hand, the cultivation of fungal species needs different culture substrates and conditions according to their group; on the other hand, certain taxonomic classes such as rust and smut fungi cannot be detected even by Polymerase Chain Reaction (PCR)-based methods using the commonly employed primers. Therefore, specific primers need to be established even for their taxonomy and phylogeny. Hence, neither culture-dependent nor culture-independent method solely can thoroughly figure out the whole structure of a given community. Because of the intrinsic selectivity of each method, the probability of a given species being detected often differs with the methods (Zhang et al. 2010). The fungal species estimated previously might be underestimated because of the estimations based on known fungi were only recorded on plants and excluded many important habitats.

The fungal species in China were also estimated by Dai and Zhuang (2010) to be about 0.18 million based on culture-dependent method. A total of 16,046 species and 297 varieties have been recorded in the Chinese territory until 2010, and the described Chinese fungal species are around 14,060 considering 10% synonyms (Dai and Zhuang 2010). By the end of 2014, the number of known fungal species in China was approximately 17,000 (Dai et al. 2015). Most of the descriptions of these species were morphology-based. From the viewpoint of this paper, a tremendous number of fungal species (probably 1.48–1.66 million) are awaiting to be discovered in China. From 2010 to 2014, a total of 912 novel species and 614 new records were described in China (Dai et al. 2015). Application of new approaches for cultures and fungal investigations should be focused on detection of new fungal taxa, especially from unusual niches such as rock habitats, cave, glaciers, etc. (Martin-Sanchez et al. 2012; Su et al. 2015; Wang et al. 2015b).

## How to detect previously undiscovered fungal species

Fungal taxonomy seeks to discover, describe and classify all species of fungi and provides tools for their identification. Specimen-based strategy has resulted in description of about 100,000 fungal species. According to the regression relationship between the numbers of described fungi and years (Figure 1), it will take centuries or millennium to describe all the fungal species on earth before being extinct. Therefore, in order to approach a complete catalogue of fungal diversity within a reasonable time frame, it is necessary to fast-track the pace of species description. However, the disadvantage of traditional morphology-based taxonomy and the massive number of active taxonomists makes it high unlikely to achieve the goal in near future (Hibbett et al. 2011).

The most common restrictions of traditional taxonomy analysis are limited taxonomic characters. Traditional biological information used for classifying fungi into major groups includes morphology, ultrastructure, physiology, tissue biochemistry, ecological traits (Wang et al. 2016) and chemotaxonomic traits (Richter et al. 2015). Phylogenetic studies have demonstrated that many morphologically similar taxa might represent distinct lineages, and numerous well-known species are in fact species complexes (Dai et al. 2015). The use of DNA sequence data to infer phylogenetic relationships among fungal lineages can help to detect cryptic species (two or more distinct species classified as a single species) with similar morphological or physiological characters. DNA barcode is a short, standardised and universal gene marker for rapid species identification of diverse groups of fungi. Species identification has been built according to DNA barcode of multiloci rather than a single locus. For example, the *Colletotrichum gloeosporioides* complex comprised of several different species with similar morphological characteristics, and it had been applied in the literature for the past 50 years. However, recently, 22 species plus one subspecies within the *C. gloeosporioides* complex were delineated using multiloci phylogenetic analyses (Weir et al. 2012). When investigating the phylogenetic diversity of *Colletotrichum* isolates associated with *Camellia* spp. using six genes, there were 11 species (including 9 well-characterised species and 2 novel species (*C. henanense* and *C. jiangxiense*) belong to the *C*. *gloeosporioides* species complex (Liu et al. 2015).

Culture-independent methods for species discovery have emerged in recent years, providing new insights into fungal diversity. The identification of some fungal groups is very difficult because they are not easily cultured, such as fungal symbionts associated with bacteria, plants and green algae, and animals including insects (Blackwell 2011), especially some nematophagous fungi in Zygomycotina. PCR-based techniques make it possible to use independent sampling methods to discover the presence of organisms without ever being seen in a culture or specimen (Blackwell 2011). The investigation on the fungal diversity associated with *O*. *sinensis* indicated that much more species were detected by PCR-SSCP analysis than culture-dependent approach (Zhang et al. 2010). When studying mycorrhizal fungi, unculturable fungi in pelotons can grow in sterile distilled water containing root extracts, but they cannot grow on artificial media (Zhu et al. 2008). So, the hyphae of these unculturable taxa can be cut out and identified using molecular technologies (Kristiansen et al. 2001).

Using a combination of environmental DNA sequencing and fluorescence microscopy, a new component of the fungal tree of life was identified and this wider group was tentatively named Cryptomycota (crypto, hidden, -mycota, phylum of fungi), which is characterised as unicellular, zoospores single-celled with a single microtubular flagellum, and cysts without a chitin/cellulose cell wall (Jones et al. 2011a). Phylogenetic analyses using multiple ribosomal RNA genes placed this clade with *Rozella*, the putative primary branch of the fungal kingdom. They differ from classical fungi in that a chitin-rich cell wall (one of the important fungal-defining characteristics) has so far not been detected (Jones et al. 2011b). However, a recent study showed that the Cryptomycota species *Rozella allomycis* does have fungal-specific chitin synthase and its resting sporangia have walls that appear to contain chitin (James and Berbee 2012). Rather than evolutionary intermediates, the Cryptomycota may be strange, divergent fungi that evolved from an ancestor with a nearly complete suite of classical fungal-specific characters.

Besides, direct sequencing of environmental DNA is a powerful tool to explore cryptic diversity of microorganisms and challenges our understanding of global biodiversity (Venter et al. 2004). A group of fungi that have lived hidden underground for millions of years only through its environmental sequences have been cultivated, classified and formally named Archaeorhizomycetes (Rosling 2011). Although their precise ecological niches and their complete life cycle remain unknown, the isolation and description of cultures of this group will allow their role in terrestrial ecosystems to be deciphered by *in vitro* characterisation and genome sequencing (Rosling et al. 2011). Similar to recently described aquatic lineage Cryptomycota, these observations ofArchaeorhizomycetes contribute towards cataloguing and understanding the missing diversity of the fungal kingdom (Hawksworth 1991).

The rapid development of automated, high-throughput methods has made it possible to acquire whole genome sequences for population-level studies (Liti et al. 2009) and has proven invaluable for investigating diverse environmental and host-associated microbial communities (Franzosa et al. 2015). Whole-metagenome shotgun (WMS) sequencing and amplicon sequencing not only reveal the fungal species in unusual environment, but also indicate the possible function of the microorganism in the environment. Time courses within communities reveal changes in response to stimuli and other dynamical properties (Franzosa et al. 2015), and thus could be applied to study the life cycle of fungal species. The shortcoming of describing fungal species through WMS sequencing is that the unique whole genome of single fungal species cannot be constructed from metagenomes; however, this problem can be solved by single-cell isolation and genome sequencing (Prosser 2015). A single-cell genome sequencing (SiC-seq) approach coupled with the fluorescence in situ hybridization (FISH) methods may provide crucial tools to describe novel, really unculturable fungal species.

To answer the question “where are the remaining fungal species to be found?”, Hawksworth and Rossman (1997) considered that these fungi reside in un-studied niches as well as known habitats explored by applying new techniques. This highlights the importance of unexplored substrata or habitats and unusual techniques. Many regions and habitats of the world need to be included in fungal discovery. “Unusual niches” are habitats where certain abiotic factor(s) imposed a condition that restricts or prevents growth of most organisms or niches normally not investigated for fungi (Cantrell et al. 2011). As indicated by the classic dictum “everything is everywhere, but the environment selects” (Beijerinck 1913), some studies showed that microorganisms also exhibit biogeographical patterns (Fierer 2008; Wu et al. 2013). Therefore, the fungal community in unusual niches may be quite different from other ecosystem to adapt the environments. For example, this concerns the “cryptoendolithic” communities immersed in rocks in Antarctica (Friedmann 1982), and the anaerobic flagellate fungi in the guts of vascular plants (Orpin 1993). In a study of 209 species of hypogeous fungi in south-eastern mainland Australia, 152 species were undescribed in previous literatures (Claridge et al. 2000). Studies on fungi in unusual niches have indicated that many unculturable fungi could be culturable on certain substrates and conditions (Singh et al. 2010). A new technique for isolating mycorrhizal fungi with pelotons has been described and this new method can increase isolation efficiency and culture the slow growing fungi (Zhu et al. 2008).

## Fungal nomenclature based on environmental sequences

During the last two decades, ecological surveys of microbial diversity using next-generation sequencing strategies have resulted in detection of huge number of unnamed molecular operational taxonomic units (MOTUs) (Hibbett et al. 2011). Little efforts to know those MOTUs extremely restricted the deep understanding of ecological functions, comparison between different studies and communications for fungal diversity. There is a pressing need to develop classification systems based on environmental sequences. Hibbett et al. (2011) proposed to assign Latin binomials or “candidate species” category to MOTUs. Recently, to permit DNA sequence data to be used as type of name for fungi was proposed as one of the modification provisions related solely to fungi in the International Code of Nomenclature for algae fungi, and plants for the discussion in the 11th International Mycological Congress (Hawksworth et al. 2018). The proposal emphasised that the new taxon based on the DNA sequence data should be described with reference to a published phylogenetic analysis but there is no more information on the new taxon (Hawksworth et al. 2018). However, this issue initiated vigorous discussion and objecting opinions from deliberations in the International Commission for the Taxonomy of Fungi and proposed 10 reasons why a sequence-based nomenclature is not useful for fungi anytime soon (Thines et al. 2018). An extensively supported viewpoint has been proposed against the proposal of Hawksworth et al. (2018), but encouraged a functional system for environmental sequences under the Candidatus or species hypotheses approach that could result from a carefully selected set of requirements to ensure high-quality data and reproducibility (Zamora et al. 2018). Although Lücking et al. (2018) responded to those augments and modified proposal, however, a recent case study on species from fungal genera *Botryosphaeria, Colletotrichum, Penicillium* and *Xylaria* showed that it is inappropriate to use mgDNA as holotypes in assigning names to fungal species due to the shorter fragments of internal transcribed spacer (ITS) sequence data obtained from environmental sequencing (Hongsanan et al. 2018).

Indeed, huge environmental DNA sequences have been detected by ecological surveys but there is no strong requirement for those studies to name those sequences because sequence data alone cannot provide biological means. However, those sequences reflect occurrence of fungal diversity that attracted fungal taxonomists to name them. In fact, majority of the environmental sequences in certain community study normally distributed in culturable group of fungi and their cultures should be obtainable. Wolfe et al. (2014) developed cheese rinds as model microbial communities by characterising *in situ* patterns of diversity and by developing an *in vitro* system for community reconstruction to bridge the gap between observations of patterns of microbial diversity and mechanisms that can explain these patterns. Therefore, it is not possible yet to determine which environmental genes originated from which genome or cell; thus, it is not possible to link phylogeny of different genes (Prosser 2015). Currently, the species delimitation of most of the fungal groups required multiloci phylogenic analysis. The development of molecular techniques can make fungal body visible *in situ* (Jones et al. 2011a, 2011b) and their single cell can be extracted and single-cell genomics can be used to expand the fungal tree of life (Ahrendt et al. 2018). If multigene phylogeny can be performed from genome DNA (gDNA) then it can be used for naming fungal species in future provided appropriate methodology is followed. Theoretically, all fungal species based on environmental sequences should have physical bodies. So, we do not need to use DNA sequences data as normal type. We propose the digital type for those DNA sequences. The digital type can be temporal supplementary before the physical type and gDNA type being available.

## Perspective on nomenclature proposal for fungal physical type, gDNA type and digital type

Numerous novel fungal species and genera have been cultured and taxonomically assigned to the nomenclature system based on morphological characters. However, use of DNA sequencing technologies allowed researchers to target and sequence conserved DNA region in fungal species to further reconfirm the existence of novel species. In several cases, morphological characters of the already reported and novel species had high similarities but only DNA sequences of specific targeted genes were used for delimiting fungal species. Based on this, we suggest that the existing fungal taxa should be categorised into physical type and gDNA type. Fungal specimens having vouchers available should be referred as the physical type. Whereas, the culturable or unculturable fungal species having complete genome sequence available should be categorised into gDNA type. Importantly, the major issues in taxonomy have been recently reported for voucher-less sequences obtained from next-generation sequencing technologies. Most of the fungal sequences obtained from these technologies often do not assign to lower taxonomic ranks due to lack of taxonomic information in databases. These taxa required scientific names to facilitate communication about them. As mentioned by Hawksworth et al. (2016) that in the current *Code* (McNeill et al. in Regnum Veg. 154. 2012), DNA sequences cannot be used solely to assign scientific names to fungal species until the physical voucher specimens or any illustrations that can act as the holotypes are available. This seemed to be against the objectives of *Code* (Pre. 1), which is specifically designed to develop a stable system for assigning scientific names to all algae, fungi and plants (Hawksworth et al. 2016). Moreover, the *Code* does not prohibit using any category of characters for separating the taxa; thereby, the data obtained from sequencing the gDNA can be used in assigning names to fungal species and acceptable as a diagnostic character (Hawksworth et al. 2016). Here, we propose that DNA sequence data generated from next-generation sequencing of environmental DNA could be permissible as digital types for fungi when no physical specimen and whole gDNA sequence are available because of any technical reason. The fungal name based on the “digital type” could be assigned as the “clade” name + species name. The “clade” name could be the name of genus, family or order, etc. which the sequence of digital type affiliates to. The digital type can be temporal supplementary before the physical type and whole gDNA type being available, but physical type should always have priority.

## How to deal with the environmental sequences for fungal nomenclature classification

Next-generation sequencing approaches have resulted in generation of numerous fungal OTUs from various habitats (Tedersoo et al. 2014; Voříšková et al. 2014; Womack et al. 2015; Zhang et al. 2016; Davison et al. 2018). The fungal OTUs retrieved from different environmental samples are basically a large set of ITS, SSU and LSU sequences obtained from the environmental gDNA (Voříšková et al. 2014; Varela‐Cervero et al. 2015; Womack et al. 2015). Although, different algorithms and threshold level are used to assign the sequences to OTUs, a research by Schmidt et al. (2014) showed that several OTU clustering approaches generally provide same OTUs across different habitats. However, most of the sequences assign to fungal OTUs are not often classified at different taxonomic ranks due to lack of taxonomic information in fungal databases. This problem let those OTUs to be considered as “unclassified” and subsequently may be ignored permanently. Theoretically, OTU sequence represents one physical fungal body in a microhabitat. However, one of the major concerns is the use of single barcoding locus for fungal community investigation such as ITS, which varies among different groups of fungi. Indeed, this is considerable point from a taxonomic point of view especially for fungi where multiple loci are required for species delimitation. Using strong analytical and correlation analysis to compare already published full-sequence of known strains with data generated from next-generation sequencing could allow us to determine the effectiveness of ITS, SSU and LSU amplicons data to discriminate species and also mark unknown OTUs as temporal supplementary before the physical type being available. Moreover, there is a need to look one step ahead and allow next-generation sequencing method coupled with current taxonomic methods to compare the diversity from a particular niche and to check the reliability of data obtained from such methods. This could provide a possibility to assign names to otherwise unknown OTUs for taxonomic studies. In a recent study using high-throughput amplicons sequencing, it has been found that the taxonomic accuracy of fungal OTUs detected from soil samples based on full-length ITS sequences was higher than ITS1 and ITS2 region of the ITS locus (Yang et al. 2018). Importantly, the multiple marker genes available to date for species delimitation could be sequence by high-throughput sequencing approach even without compromising the sequence length as it has shown that the near full-length 16S rRNA amplicons could be sequenced on Illumina Miseq sequencer (Burke and Darling 2016).

Recent methodological advancement in genomics such as SiC-seq has enabled to sequence large population including up to 50,000 cells per run. This approach uses droplet microfluidics to isolate, fragment and barcode the genomes of single cells, followed by Illumina sequencing of pooled DNA (Lan et al. 2017). The nucleotide sequence of marker genes required for species delimitation can be extracted from fungal genome and used to identify and assign names to novel phylogenetic lineages and fungal taxa. Moreover, this genomic approach will also provide information on functional role of the microbes in specific niche. Most of the microbes are soil-inhabiting and the development of iChips has improved culturing techniques to culture previously uncultivable species from soil (Nichols et al. 2010). These techniques could inspire a strategy to increase the pool of currently cultivable fungi, and subsequently help to identify the unknown species and replace digital type with physical type.

## Concluding remarks

“How many fungal species occur on our planet” is an attractive question for mycologists and general public. Previous estimations were primarily based on an average ratio of numbers of fungi recorded on particular plants. Recently, culture-independent approaches, especially large-scale environmental sequencing method, have provided new insight to estimate the numbers of fungi (Blackwell 2011). Compared with the well-accepted estimation of 2.2–3.8 million culturable fungal species, the statistical ratio (1:8.8) of the numbers of cultured fungi and the OTUs detected each of the same substrates from published literatures were deduced. Therefore, around 12 million fungal species on earth were estimated, which is far more than the previous estimations (O’Brien et al. 2005; Blackwell 2011; Hawksworth 2012; Hawksworth and Luecking 2017). However, the connection between fungal species and OTUs is challengeable and needs to be answered by mycological community. Herewith, we propose that attempts to get the cultures of specimens as physical type are the priority, the complete genome sequence of fungi (single-cell genome) as gDNA type is the second choice, and fungal sequence data generated from environmental genomic DNA can temporary be as digital type. We could assign the fungal name informally to the OTU (the digital type) belong to the clade (any taxa level above species) and species name, which should be useful for comparison and communication between different studies. However, the new assessment on fungal species calls for more knowledge on fungal inhibiting environments and the use of new molecular approaches. Therefore, cooperation and communication of mycologists all over the world is crucial for the study of fungal diversity.
